# Characterization of the Rate-Limiting Steps in the Dark-To-Light Transitions of Closed Photosystem II: Temperature Dependence and Invariance of Waiting Times during Multiple Light Reactions

**DOI:** 10.3390/ijms24010094

**Published:** 2022-12-21

**Authors:** Melinda Magyar, Gábor Sipka, Wenhui Han, Xingyue Li, Guangye Han, Jian-Ren Shen, Petar H. Lambrev, Győző Garab

**Affiliations:** 1 Institute of Plant Biology, Biological Research Centre, 6726 Szeged, Hungary; 2 Photosynthesis Research Center, Key Laboratory of Photobiology, Institute of Botany, Chinese Academy of Sciences, Beijing 100093, China; 3 Research Institute for Interdisciplinary Science, and Graduate School of Natural Science and Technology, Okayama University, Okayama 700-8530, Japan; 4 Faculty of Science, University of Ostrava, 710 00 Ostrava, Czech Republic

**Keywords:** chlorophyll-*a* fluorescence, conformational changes, dielectric relaxation, light-adapted charge-separated state of PSII, rate-limitation, temperature-dependence, waiting time

## Abstract

Rate-limiting steps in the dark-to-light transition of Photosystem II (PSII) were discovered by measuring the variable chlorophyll-*a* fluorescence transients elicited by single-turnover saturating flashes (STSFs). It was shown that in diuron-treated samples: (i) the first STSF, despite fully reducing the Q_A_ quinone acceptor molecule, generated only an *F*_1_(<*F*_m_) fluorescence level; (ii) to produce the maximum (*F*_m_) level, additional excitations were required, which, however, (iii) were effective only with sufficiently long Δ*τ* waiting times between consecutive STSFs. Detailed studies revealed the gradual formation of the light-adapted charge-separated state, PSII_L_. The data presented here substantiate this assignment: (i) the Δ*τ*_1/2_ half-increment rise (or half-waiting) times of the diuron-treated isolated PSII core complexes (CCs) of *Thermostichus vulcanus* and spinach thylakoid membranes displayed similar temperature dependences between 5 and –80 °C, with substantially increased values at low temperatures; (ii) the Δ*τ*_1/2_ values in PSII CC were essentially invariant on the *F*_k−_to-*F*_k+1_ (*k* = 1–4) increments both at 5 and at −80 °C, indicating the involvement of the same physical mechanism during the light-adaptation process of PSII_L_. These data are in harmony with the earlier proposed role of dielectric relaxation processes in the formation of the light-adapted charge-separated state and in the variable chlorophyll-*a* fluorescence of PSII.

## 1. Introduction

In this paper we investigate special properties of the recently discovered rate-limiting steps in Photosystem II (PSII) [1,2]. PSII is a multi-subunit pigment-protein complex embedded in the thylakoid membranes (TMs) of plants, algae, and cyanobacteria. It uses light energy to catalyze the electron transfer from water to plastoquinone and supplies the reducing equivalents necessary to fix CO_2_. PSII is probably the most-studied light-induced enzyme, not only for its relevance to biochemistry, being the only water-splitting and O_2_-producing enzyme, but also because it serves as a source of inspiration for artificial photocatalysis to produce H_2_.

The structure and the primary photophysical and photochemical functions of PSII are well known [3,4,5,6]. The core complex (CC) of PSII contains the reaction center (RC) incorporated in the D1/D2 proteins, the α and β subunits of cytochrome *b*_559_, two integral antenna proteins, CP43 and CP47, and the oxygen-evolving complex (OEC) [7]. The trapping of light energy and its transformation into electrochemical free energy occurs within the RC complex [8]. In open-state PSII (PSII_O_), upon illumination, a P_680_^+^/Pheo^−^ radical pair is formed during the primary photochemical reaction, in several picoseconds due to the electron transfer from the primary electron donor P_680_ to pheophytin (Pheo). Subsequent electron transfer steps – from Pheo^−^ to Q_A_, the first quinone electron acceptor, and from the tyrosine residue (Y_Z_) on the D1 protein to P_680_^+^, followed by the oxidation of the Mn_4_CaO_5_ cluster, leading to S_2_ state of the OEC – stabilize the charge separated state. PSII with all Q_A_ reduced is considered the closed state of PSII (PSII_C_).

The generation of the stable charge separation in PSII is followed by somewhat slower electron and proton transfer reactions at the acceptor and donor sides, between Q_A_ and Q_B_, the primary and secondary quinone acceptors, and in the OEC, respectively. In TMs, the linear electron-transport chain, via the cytochrome *b*_6_*f* complex and PSI, supply electrons to the terminal electron acceptor CO_2_. Evidently, the continual operation of the electron transport requires the repeated generation of charge separation in PSII, which can occur only after re-opening the RC. The re-opening time of the PSII RC is determined by the rates of the secondary electron transfer reactions, which occur on timescales between a hundred microseconds and a millisecond [9,10]. Additional limitations in the operation of the PSII electron transfer reactions, and thus in the re-opening time of the RC, are imposed by the relatively slow (5–10 ms) charge transfer reactions of the cytochrome *b*_6_*f* complex [11]. Because of this rate-limiting step in the electron-transport system, under continuous illumination, PSII RC may be found with a high probability in a closed state, especially at high light intensities. The effective turnover time of the electron-transport system might be further increased under inorganic carbon limiting conditions, which can hinder the operation of the photosynthetic electron transport [12,13]. For this reason, the effect of illumination on PSII_C_ is of substantial interest.

In recent years, our understanding of the light-induced structural dynamics of PSII advanced substantially. It is now well established that the secondary electron and proton transfer events are associated with well-discernible reorganizations both on the donor and the acceptor sides. Time-resolved serial femtosecond crystallography experiments using X-ray free electron lasers revealed structural changes in PSII CC of *Thermostichus* (*Thermosynecococcus*) (*T.*) *vulcanus*—around the Q_B_/non-heme iron and the Mn_4_CaO_5_ cluster [14,15,16]. Light-induced reorganizations around the Q_B_ pocket have also been shown to occur in purple-bacterial reaction centers (bRCs) [17,18,19]. The crystal structure of PSII RC shows large similarities to bRC, its purple-bacterial ancestor [20,21,22,23].

In our recent work [2], using FTIR spectroscopy to monitor the kinetics of charge-recombination S_2_^(+)^Q_A_^–^ → S_1_Q_A_ in *T. vulcanus* PSII CC, we observed a three-fold increase in the lifetime of PSII_C_ upon exposing them to a train of 20 single-turnover saturating flashes (STSFs); PSII_C_ was generated by the first STSF of the train. The stabilization of the charge-separated state was attributed to the gradual formation of PSII_L_, the charge-separated light-adapted state of PSII. Similar, but much more pronounced stabilizations of the charge-separated state were observed earlier in bRCs upon continuous illumination of the RC complexes [24,25,26]. These transitions, which were reminiscent of the Kleinfeld effect [27], were ascribed to conformational memory of bRC proteins and the formation of their light-adapted charge-separated state [28,29,30,31,32].

We also recorded variable chlorophyll-*a* (Chl-*a*) fluorescence transients (*F*_v_) elicited by trains of STSFs on diuron-treated isolated plant TMs and PSII CC of *T. vulcanus*. (*F*_v_ = *F*_m_ − *F*_o_, where *F*_m_ and *F*_o_ are the maximum and the minimum fluorescence levels, respectively; *F*_o_ is associated with PSII_O_; diuron, DCMU, and 3-(3,4-dichlorophenyl)-1,1-dimethylurea inhibits the inter-quinone electron transfer in PSII and allows only one stable charge separation. In accordance with Joliot and Joliot [33], we found that the fluorescence yield after the first STSF, which leads to the reduction of Q_A_, produces only an intermediate *F*_1_ level, and additional STSFs were required to reach the maximum fluorescence level (*F*_m_). We also found, however, a peculiar feature of these transients of *F*_v_: to induce sizeable increments from the *F*_1_ level to the *F*_2_ level, relatively long Δ*τ* waiting times must be allowed between STSFs, revealing rate limitations in this process [1]. It is to be emphasized that the second and consecutive flashes, which induce the *F*_1_-to-*F*_2_, *F*_2_-to-*F*_3_ etc. fluorescence increments, do not generate any further stable charge separation, i.e., PSII_C_ is generated by the first STSF, which produces the *F*_1_(<*F*_m_) fluorescence level [1,2,33,34,35]. It has also been clarified that the rate limitations do not arise from gating of the primary photochemistry: in DCMU-treated PSII CC of *T. vulcanus*, additional excitations, after the generation of the stable charge separation by the first STSF, produce only rapidly recombining P_680_^+^Pheo^−^ radical pairs, with recombination rates orders of magnitude faster than the Δ*τ_1/2_* half-waiting times [36]. Based on these features and the strong similarity of the light-adapted states in bRC and in PSII, we adopted the explanation offered for the light-induced stabilization of the charge-separated state of bRC [37,38]. Accordingly, the light-induced formation of PSII_L_ was proposed to be associated with conformational changes and dielectric relaxation processes, possibly combined with the effects of local heat packages [1,2].

To understand the nature and physical mechanisms of these waiting-time-related processes, and thus also the origin of *F*_v_, which carries important information on the functional activity and structural dynamics of PSII [39,40,41], systematic investigations are required. We have already examined the effect of the lipidic environment of PSII and revealed the shortening of Δ*τ*_1/2_, from ~1 ms to ~0.2 ms, upon the addition of plant TM lipids to isolated *T. vulcanus* PSII CC; Δ*τ*_1/2_ values in intact *T. vulcanus* cells were comparable to those in plant TMs [42]. These data have shown that the processes underlying the light-induced transition of PSII_C_ to PSII_L_ depend significantly on the lipid content of the RC matrix. In general, these data also suggest the role of physicochemical factors in the RC complexes. Here, we studied the temperature dependence of the Δ*τ*_1/2_ half-waiting times in isolated PSII CC of *T. vulcanus* and in spinach TMs. We also tested the possible dependence of Δ*τ*_1/2_ on the number of STSFs applied. We found that: (i) although the Δ*τ*_1/2_ values in PSII CC are considerably larger than in TMs, their temperature dependences follow a very similar pattern, with substantially increased Δ*τ*_1/2_ values at low temperatures; and (ii) Δ*τ*_1/2_ appeared to be essentially invariant on the *F*_k_-to-*F*_k+1_ fluorescence increments (*k* = 1–4), indicating the involvement of the same process during the light-adaptation of closed PSII RC.

## 2. Results and Discussion

To characterize the gradual light-induced formation of the charge-separated light-adapted state (PSII_L_) from its closed state (PSII_C_) and to gain information on the underlying physical mechanism, we investigated the STSF-induced Chl-*a* fluorescence increments in isolated PSII CC of *T. vulcanus* and spinach TMs in the presence of DCMU, which keeps the reaction centers in closed state, being capable of accepting only one electron. Under our experimental conditions, in the temporal interval of interest, the PSII_C_-to-PSII_O_ via charge recombination can be neglected.

### 2.1. Temperature Dependence of the Variable Chl-a Fluorescence (F_v_) Induced by STSFs

**Figure 1:**
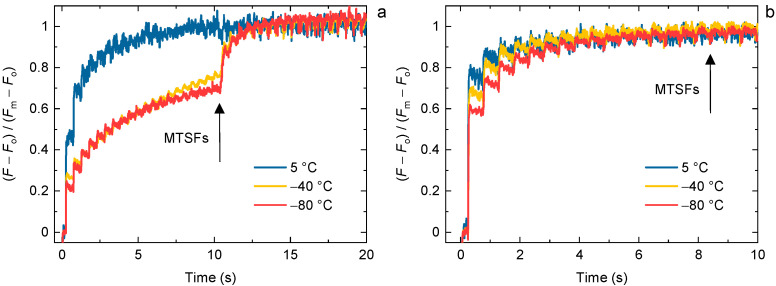
Temperature-dependent variations of the single-turnover saturating flash (STSF) induced Chl-*a* fluorescence transients of DCMU-treated PSII CC of *T. vulcanus* (**a**) and spinach TMs (**b**). The STSFs were applied 500 ms apart; at the end, blue laser flashes (multiple-turnover saturating flashes, MTSFs) with different lengths and amounts were fired to ensure the saturation. The measurements were performed using the PAM-101 based setup.

Upon the excitation of DCMU-treated dark-adapted PSII containing samples—PSII CC of *T. vulcanus* and spinach TMs—by trains of STSFs stepwise increments of the *F*_v_ Chl-*a* fluorescence rise were observed (Figure 1), in accordance with our earlier data [1,2]. It is also shown that both the *F*_1_ level and the number of STSFs required to reach *F*_m_ depended strongly on the temperature: the *F*_1_ levels gradually decreased while the required number of STSFs gradually increased upon the stepwise decrease in the temperature. With reasonable agreement with our earlier observations [1], the *F*_1_ level in PSII CC at −80 °C did not exceed 25–30% of *F*_m_; at 80 K, this value was <15% [2]. In TMs, the decrease in the *F*_1_ level at low temperatures was less marked (at −80 °C ~60% of *F*_m_), but still well discernible. Similar differences between the two samples were seen in the number of STSFs to reach *F*_m_. These differences might originate from variances in the molecular composition between our cyanobacterial and plant PSII samples with different rigidities. In general, proteins from thermophilic organisms possess higher dynamical stiffness than from mesophilic organisms [43]. This might explain the lower conformational adaptation of our PSII CC when compared to TMs obtained from the thermophilic *T. vulcanus* cells and the mesophilic spinach leaves.

### 2.2. Temperature Dependence of Δτ_1/2_

**Figure 2:**
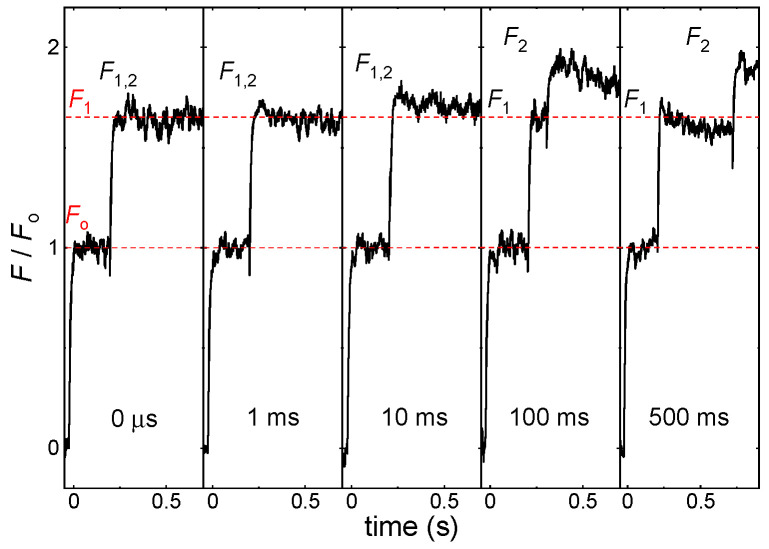
Kinetic traces of Chl-*a* fluorescence transients of DCMU-treated PSII CC of *T. vulcanus* at −80 °C; the traces were elicited by double STSFs fired with the indicated time intervals between the two flashes. *F*_1,2_ marks the fluorescence levels after the double STSFs and, where resolved, *F*_1_ and *F*_2_ show, respectively, the levels reached after the first and the second STSF. The measurements were performed using the PAM-101 based setup.

Figure 2 illustrates the peculiar feature of the *F*_1_-to-*F*_2_ fluorescence increments in dark-adapted DCMU-treated *T. vulcanus* PSII CC at −80 °C. It shows a strong dependence of the magnitude of the fluorescence increment on the Δ*τ* waiting time between the first and the second STSF. This phenomenon has already been demonstrated on *T. vulcanus* PSII CC, whole cyanobacterial cells, spinach TMs [1], and in samples with different lipid compositions [42]. As discussed above, in the presence of DCMU, after the first STSF Q_A_ is reduced, and the second STSF induces no further stable charge separation. Nevertheless, after a sufficiently long Δ*τ* dark waiting time the fluorescence level elicited by the second STSF increases (Figure 2). While these data are similar to those reported earlier on PSII CC at room temperature [36], they reveal strikingly longer Δ*τ*_1/2_ values at −80 °C. This prompted us to investigate the temperature dependences of Δ*τ*_1/2_ in *T. vulcanus* PSII CC and spinach TMs.

**Figure 3:**
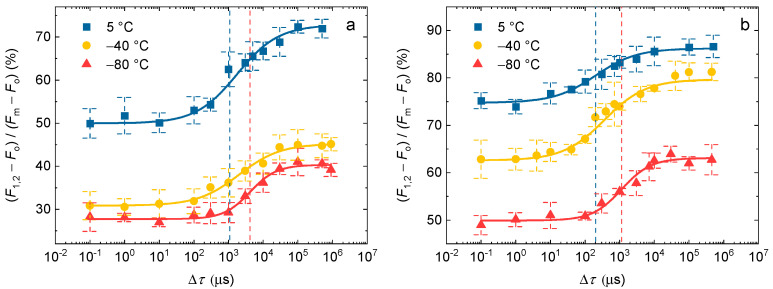
Dependence of the *F*_1_-to-*F*_2_ Chl-*a* fluorescence levels on the Δ*τ* time intervals between the first and second STSFs at different temperatures in PSII CC of *T. vulcanus* (**a**), and in spinach TMs (**b**) in the presence of 40 μM DCMU. Continuous lines represent logistic-function fits of the data points, which are shown as mean values ± SD (*n* = 3–9). Dotted vertical lines mark the Δ*τ*_1/2_ half-rise time values, i.e., the Δ*τ* values corresponding to the 50% of the maximum *F*_1_-to-*F*_2_ increments at 5 °C (blue) and −80 °C (red). The fluorescence levels at each Δ*τ* were determined after the second STSF; here marked as *F*_1,2_, irrespective of the resolution of the *F*_1_ and *F*_2_ levels (see Figure 2). The measurements were performed on the PAM-101 based setup.

To determine the temperature dependence of ∆*τ*_1/2_ half-waiting times between the first and the second STSFs, we investigated the double-STSF induced transients on DCMU-treated PSII CCs of *T. vulcanus* and spinach TMs at distinct temperatures between 23 and −80 °C, with a broad range of ∆*τ* waiting times between the two STSFs. Note that in Figure 3, we mark the increment induced by the second-STSF as *F*_1,2_, irrespective of whether or not the *F*_1_ and *F*_2_ fluorescence levels were resolved at the applied time resolution of the fluorimeter (cf. Figure 2). The half-rise (or half-waiting) times (∆*τ*_1/2_) of the *F*_1_-to-*F*_2_ increments were obtained from a logistic-function fit of the dependence of the fluorescence increments on ∆*τ* (Figure 3). Table 1, in addition to the ∆*τ*_1/2_ values, contains data on the *P* parameters (steepness) of the logistic functions, as well as on the *F*_v_/*F*_m_ parameters, which characterize the photochemical activity and structural dynamics of PSII [2]. The *F*_v_/*F*_m_ values in PSII CC were very similar to those obtained in our earlier studies [2,36]; in TMs, they were somewhat lower than usual, also in the intact leaves used. Nevertheless, the ∆*τ*_1/2_ values at room temperature were very similar in all the TM preparations with similar or higher *F*_v_/*F*_m_ values [42].

The effect of rate-limitation can clearly be seen in both samples and at all temperatures. In PSII CC the ∆*τ*_1/2_ of ~1.2 ms at room temperature (RT) was comparable to those determined in our earlier studies under similar experimental conditions [42]; the same is true for the ∆*τ*_1/2_ (0.2 ms) of TM [42]. Despite the relatively large error bars, due to the small increments and the error of the fits, it is clear that the ∆*τ*_1/2_ values are significantly larger at low temperatures both in PSII CC and TMs (Figure 3); this increase in the half-waiting times is 3–5 fold in the two samples (Table 1). These data strongly suggest the involvement of the same process, despite the different values at non-cryogenic temperatures (Table 1). An interesting feature of these ∆*τ* dependent increments are that, as also reflected by increased P values, the rise appeared to be steeper at −80 °C in PSII CC and at −60 and −80 °C in TM than at higher temperatures. The origin of this difference is unclear; it might be correlated with the fact that the increments at low temperatures originate from different phases of *F*_v_ and may contain different elements of the structural dynamics of PSII.

**Figure 4:**
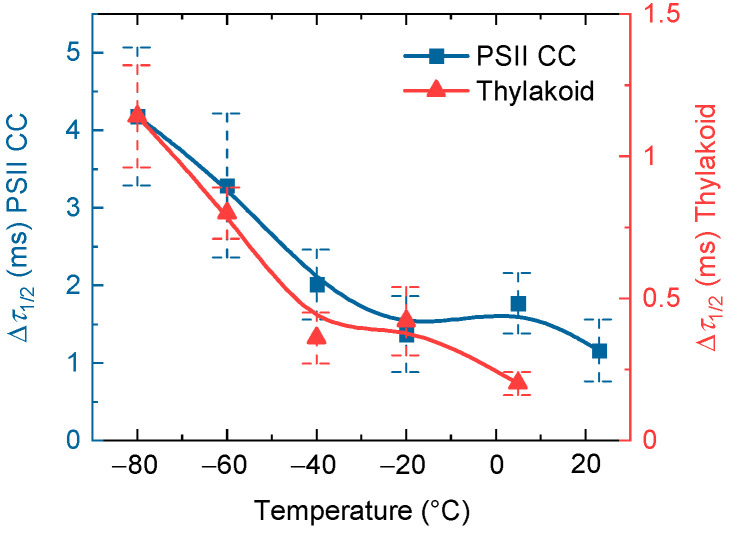
Temperature dependence of the double-STSF induced (*F*_1_-to-*F*_2_) ∆*τ*_1/2_ half-waiting times of isolated *T. vulcanus* PSII CC (blue) and spinach TMs (red). The data points are taken from Table 1. The continuous lines represent spline interpolation of the data points.

For an easier comparison of the patterns of the changes of Δ*τ*_1/2_ at different temperatures in PSII CC and TMs, we plotted the temperature dependences of the two samples on different scales (Figure 4). These data show that the variations of Δ*τ*_1/2_, despite the substantial differences at all temperatures, follow essentially the same pattern—suggesting the involvement of identical or very similar physical mechanism(s). An interesting observation is that both curves appear to possess a “breakpoint”, which can be discerned at −20 °C for PSII CC and −40 °C for TMs. The presence of these breakpoints is proposed to originate from protein phase transitions. To support this hypothesis we invoke the works of Garbers and coworkers, who, by using Mössbauer spectroscopy at cryogenic temperatures on PSII-enriched membranes, observed “the onset of fluctuations between conformational substates of the protein matrix at around 230 K” [44]. Furthermore, Pieper and coworkers, by using neutron scattering, found a “softening” of the protein matrix in the temperature range above 240 K [45,46]. The difference between the breakpoints in PSII CC and TMs can be attributed to their different growth temperatures, which, as pointed out above, might determine the conformational rigidity of the sample.

It is also worth pointing out that the mobility of protein residues might modulate the temperature dependence of the Δ*τ*_1/2_ half-waiting times. It was shown in hydrated proteins that dielectric relaxation processes occur with different lifetimes and dominance at different temperature intervals [47]. In addition to the roles of protein residues the mobility of different water molecules in the RC matrix [41,48], either on the acceptor side [49] or the donor side [50] of the RC, might also contribute to the temperature-dependent variations of Δ*τ*_1/2_.

### 2.3. Temperature Dependence of Δτ_1/2_ during Multiple Light Reactions

**Figure 5:**
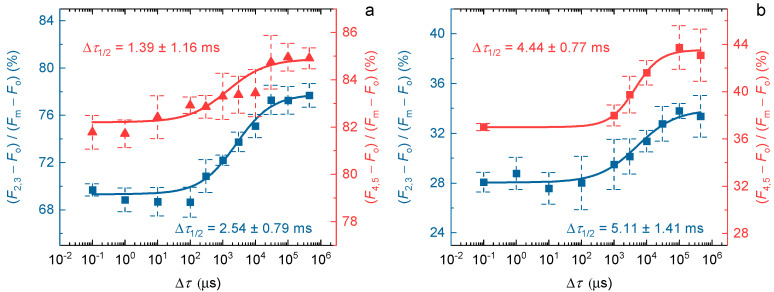
Dependence of the *F*_2_-to-*F*_3_ (blue) and the *F*_4_-to-*F*_5_ (red) Chl-*a* fluorescence levels on the ∆*τ* time intervals between the second and third, and between the fourth and fifth STSFs, respectively, in DCMU-treated *T. vulcanus* PSII CC at 5 °C (**a**) and at −80 °C (**b**). Continuous lines represent logistic-function fits of the data points, which represent mean values ± SD (*n* = 3–4); the calculated ∆*τ*_1/2_ values are also indicated. The measurements were performed on the MC-PAM based setup.

In our earlier work, we have shown that the rate-limiting step was present not only in the *F*_1_-to-*F*_2_ fluorescence increment but also between later steps [1]. However, it was not clarified whether or not the ∆*τ*_1/2_ half-waiting times depend on the number of flashes during the train of STSFs. To answer this question, we determined the ∆*τ*_1/2_ values in PSII CC for the *F*_2_-to-*F*_3_ and the *F*_4_-to-*F*_5_ increments at 5 and −80 °C (Figure 5). As shown by these measurements, only minor variations of Δ*τ*_1/2_ can be seen. At 5 °C the half-rise time of the waiting time was ~2.5 ms after the second flash and ~1.4 ms after the fourth flash (for comparison, Δ*τ*_1/2_ after the first flash was ~1.8 ms). At −80 °C Δ*τ*_1/2_ values for the *F*_2_-to-*F*_3_ and *F*_4_-to-*F*_5_ increased to ~5.1 ms and ~4.4 ms, respectively; for *F*_1_-to-*F*_2_ Δ*τ*_1/2_ was ~4 ms. One can notice that the standard deviation of the data points of later steps are higher, which is the consequence of the gradually smaller increments after each flash, thus hampering the determination of the precise fluorescence levels. Nevertheless, it can be safely concluded that the half-waiting times do not differ significantly along the grades of *F*_v_, suggesting the involvement of the same physical mechanism.

## 3. Materials and Methods

### 3.1. Growth Conditions

A thermophilic cyanobacterial strain, *Thermostichus (Thermosynechococcus) vulcanus*, isolated from a hot spring in Yunomine, Japan [51] was grown photoautotrophically in BG11 medium (pH 7.0) as a batch culture. Cells were grown at 50 °C under continuous illumination with a white fluorescent lamp at a photon flux density of 50–100 µmol photons m^−2^ s^−1^ [52], and aerated on a gyratory shaker operating at 100 rpm.

### 3.2. Sample Preparation

TMs were isolated from fresh market spinach (*Spinacia oleracea*) leaves essentially as described earlier [53], with minor modifications. Briefly, deveined leaves were homogenized in 50 mM Tricine (pH 7.5), 400 mM sorbitol, 5 mM KCl, and 2 mM MgCl_2._, and then filtered through a nylon mesh, the resulting supernatant was centrifuged then for 7 min at 6000× *g*. The pellet was resuspended in 50 mM Tricine (pH 7.5), 5 mM KCl, and 5 mM MgCl_2_, followed by the immediate addition of a buffer containing 50 mM Tricine (pH 7.5), 800 mM sorbitol, 5 mM KCl, and 2 mM MgCl_2_ before centrifugation for 7 min at 6000× *g*. The pellet was finally resuspended in 50 mM Tricine (pH 7.5), 400 mM sorbitol, 5 mM KCl, and 2 mM MgCl_2_ and stored in liquid nitrogen at a Chl concentration of 2–3 mg mL^−1^, until use.

PSII CCs of *T. vulcanus* were isolated as described earlier [54,55,56] and were diluted in a reaction buffer containing 5% glycerol, 20 mM MES (pH 6.0), 20 mM NaCl, and 3 mM CaCl_2_.

### 3.3. Chl-a Relative Fluorescence Yield Measurements

Relative fluorescence yields were measured using a PAM-101 (Pulse Amplitude Modulation) fluorometer and a Multi-Color (MC) PAM (Walz, Effeltrich, Germany). Fluorescence increments of the samples were induced by STSFs (Xe flashes, Excelitas LS-1130-3 Flashpac with FX-1163 Flashtube with reflector, Wiesbaden, Germany) of 1.5-µs duration at half-peak intensity. When using trains of STSFs, the flashes were applied 500 ms apart. The frequency of the modulated measuring light (low intensity and nonactinic) was 1.6 kHz in the case of PAM-101, while in the case of the MC-PAM it was 1 kHz. To improve the accuracy of the determination of the STSF-induced increments of the fluorescence levels, we increased the signal-to-noise of the measurement by switching the frequency of the measuring light to 100 kHz 10 ms before the flash, for 50 ms with PAM-101, and 2 ms before the flash, for 20 ms with MC-PAM. With this limitation, the actinic effect of the 100 kHz measuring beam, causing a slow rise of the fluorescence level, remained smaller than 5% for the detected intensities; this was corrected by extrapolating the fluorescence level to t = 0 of firing the STSF. The time resolutions applied were ~20 ms and ~0.13 ms, respectively, for PAM-101 and MC-PAM. With these settings, we determined the quasi steady-state fluorescence levels, and did not record the fast-rising components of the variable fluorescence (cf. [57]).

The sample was placed on the sample holder of a thermoluminescence apparatus in order to control the temperature. The timing of the flashes was controlled using a home-designed programmable digital pulse generator. In the case of the PAM-101, the kinetic traces were recorded using a National Instrument data acquisition device (DAQ 6001, Austin, TX, USA) via a custom-designed LabVIEW software; in case of MC-PAM*,* the program’s own software was used.

For Chl-*a* fluorescence transient measurements, the Chl concentration of the TMs were diluted to ~20 µg mL^−1^ in the resuspension buffer, and that of the PSII CC to ~5 µg mL^−1^ when performing double- or multiple-STSFs with variable time intervals between flashes, and to ~20 µg mL^−1^ when measuring STSF-induced fluorescence steps. DCMU was dissolved in dimethyl sulfoxide and added to all samples immediately before the fluorescence measurements at a final concentration of 40 µM (the final dimethyl sulfoxide concentration did not exceed 1%). Before the measurements, the samples were dark adapted for 5 min at room temperature, then cooled to the required temperature and were then temperature adapted for 5 more min.

## 4. Conclusions

The major goal of this study was to provide data to aid the better understanding of the mechanism(s) underlying the gradual formation of the light-adapted state (PSII_L_) from the charge-separated (closed) state (PSII_C_). As pointed out in the Introduction, this is a physiologically important process: (i) because PSII_C_ can often receive excitations, and (ii) because the PSII_C_-to-PSII_L_ leads to the stabilization of the charge-separated state; further (iii) as suggested by earlier studies, both in PSII and bRC, the process of light adaptation reflects subtle reorganizations, structural dynamics, and conformational memory in Type II RC matrices [35,41].

Here, we investigated the key features of the variable Chl-*a* fluorescence (*F*_v_) induced by trains of STSFs in DCMU-treated isolated PSII CC of *T. vulcanus* and spinach TMs. In particular, we were interested in the basic peculiar characteristics of *F*_v_, its dependence on the waiting times (Δ*τ*) between excitations; to obtain significant magnitudes of consecutive STSF-induced fluorescence increments along *F*_v_, sufficiently long Δ*τ* values are required [1]. Earlier studies have shown that the Δ*τ*_1/2_ half-waiting times (where the *F*_1_-to-*F*_2_ fluorescence increment reaches 50% of its maximum) depend on the lipidic environment of the RC matrix [42]—suggesting determining roles of physicochemical factors in Δ*τ*_1/2_.

Here, we show (i) that the Δ*τ*_1/2_ values in PSII CC and spinach TMs display a similar pattern of temperature dependences between 5 and −80 °C, with increased values at low temperatures; and (ii) that the Δ*τ*_1/2_ values in PSII CC are essentially invariant on *k* (*k* = 1–4), denoting the *F*_k_-to-*F*_k+1_ increments, both at 5 and at −80 °C. These data strongly suggest that the underlying physical mechanisms are essentially the same during these processes. In line with earlier conclusions [2,38], we propose that the formation of the light-adapted charge-separated states in bRC and PSII depend largely on dielectric relaxation processes. The protein matrix of PSII seems to reach the optimal dielectric environment gradually by additional excitations, a process that is significantly hindered at low temperatures. Recent studies have shown the involvement of a network of hydrogen bonds around some protein residues and bound water molecules in bRC [58]; bound-water containing domains on the donor side of PSII RC might play critical roles in the dielectric relaxation processes, and thus also in the variable Chl-*a* fluorescence [41].

## Figures and Tables

**Table 1:** Double-STSF induced (*F*_1_-to-*F*_2_) Δ*τ*_1/2_ half-rise times of PSII CC of *T. vulcanus* and spinach TMs in the presence of DCMU at different temperatures. Measurements were performed, using the PAM-101 based setup, on the same PSII CC batch and the same TM preparations at all temperatures. *P* is the slope of the rise curve calculated by logistic-function fit of the data points, which represent mean values ± SD (*n* = 3–9). Numbers marked with the symbol * are obtained from a global fit with shared *P* (0.73); at low temperatures (≤−60 °C), the global fit was not satisfactory, and we allowed free run of the fit. The *F*_v_/*F*_m_ parameter values are also shown.

*Temperature* *(°C)*	*PSII CC* *Δτ_1/2_ (ms)*	*Logistic Fit P*	*F_v_/F_m_*	*Thylakoid* *Δτ_1/2_ (ms)*	*Logistic Fit P*	*F_v_/F_m_*
23	1.16 ± 0.40	0.73 *	0.80 ± 0.01	-	-	-
5	1.77 ± 0.39	0.73 *	0.85 ± 0.02	0.20 ± 0.04	0.73 *	0.65 ± 0.00
−20	1.37 ± 0.49	0.73 *	0.83 ± 0.01	0.42 ± 0.12	0.73 *	0.60 ± 0.01
−40	2.01 ± 0.45	0.73 *	0.83 ± 0.01	0.36 ± 0.09	0.73 *	0.56 ± 0.01
−60	3.29 ± 0.93	0.73 *	0.83 ± 0.00	0.80 ± 0.09	1.3	0.53 ± 0.02
−80	4.18 ± 0.89	1.15	0.83 ± 0.01	1.14 ± 0.18	1.03	0.50 ± 0.02

## Data Availability

All data supporting the findings of this study are available from the corresponding author upon request.
